# Identification of a Transcriptional Prognostic Signature From Five Metabolic Pathways in Oral Squamous Cell Carcinoma

**DOI:** 10.3389/fonc.2020.572919

**Published:** 2020-12-02

**Authors:** Xiang Wu, Yuan Yao, Zhongwu Li, Han Ge, Dongmiao Wang, Yanling Wang

**Affiliations:** ^1^Jiangsu Key Laboratory of Oral Disease, Nanjing Medical University, Nanjing, China; ^2^Department of Oral and Maxillofacial Surgery, Affiliated Hospital of Stomatology, Nanjing Medical University, Nanjing, China

**Keywords:** oral squamous cell carcinoma, metabolic pathways, prognostic signature, single sample gene-set enrichment, LASSO

## Abstract

Dysregulated metabolic pathways have been appreciated to be intimately associated with tumorigenesis and patient prognosis. Here, we sought to develop a novel prognostic signature based on metabolic pathways in patients with primary oral squamous cell carcinoma (OSCC). The original RNA-seq data of OSCC from The Cancer Genome Atlas (TCGA) project and Gene Expression Omnibus (GEO) database were transformed into a metabolic pathway enrichment score matrix by single-sample gene set enrichment analysis (ssGSEA). A novel prognostic signature based on metabolic pathways was constructed by LASSO and stepwise Cox regression analysis in the training cohort and validated in both testing and validation cohorts. The optimal cut-off value was obtained using the Youden index by receiver operating characteristic (ROC) curve. The overall survival curves were plotted by the Kaplan-Meier method. A time-dependent ROC curve analysis with 1, 3, 5 years as the defining point was performed to evaluate the predictive value of this prognostic signature. A 5-metabolic pathways prognostic signature (5MPS) for OSCC was constructed which stratified patients into subgroups with favorable or inferior survival. It served as an independent prognostic factor for patient survival and had a satisfactory predictive performance for OSCC. Our results developed a novel prognostic signature based on dysregulated metabolic pathways in OSCC and provided support for aberrant metabolism underlying OSCC tumorigenesis.

## Introduction

Oral squamous cell carcinoma (OSCC) is a common malignant tumor in the head and neck region which is closely associated with smoking and alcohol abuse (1). Currently, it is frequently treated with a combination of surgery, radiotherapy and chemotherapy in the clinic. Despite significant advances in clinical management of OSCC, its prognosis still remains poor over the past years (2). The reasons for unfavorable prognosis might be due to limited understanding of genetic, molecular and metabolic events underlying oral tumorigenesis as well as the lack of effective prognostic predictors and therapeutic targets (3). Furthermore, these traditional prognostic indicators such as tumor size, margin status and tumor stage remain far from optimal and usually fail to meet clinical needs due to substantial survival variations in patients within the same catalogs (2, 4). Thus, it is necessary and urgently required to identify new, effective, sensitive biomarkers for early detection, diagnosis and prognosis of OSCC.

The intricate link between dysregulated metabolism and tumorigenesis has been increasingly appreciated. Metabolic reprogramming such as glycolysis has been identified as a well-established hallmark of cancer (5). Altered metabolic pathways in cancer have become a driving force for cancer cells to gain beneficial energy or evade immune surveillance, thus suggesting that these changes can be exploited for biomarkers and therapeutic targets developments (6). Indeed, dysregulation of individual or multiple metabolic pathways has been explored as diagnostic or prognostic biomarkers across several human cancers. Several metabolic signatures at the transcriptional level have been reported to predict patients’ survival in hepatocellular carcinoma and ovarian cancer (7, 8). However, most of these studies usually focused on the prognostic signatures based on genes involved in a single metabolic pathway, while some other metabolic abnormalities in cancer might be neglected. Thus, an integrative signature based on multiple metabolism-related gene sets might be better to capture the full metabolic dysregulations in cancer and have superior performance in prediction. Comprehensive analyses of metabolic pathways in head and neck squamous cell carcinoma (HNSCC) have yielded predictive models and established prognostic predictors with high performance (9). However, HNSCC is a heterogeneous group of epithelial malignancies with diverse etiologic factors, tumorigenic processes and treatment modalities, as exemplified by HPV infection and its distinct roles between OSCC and oropharyngeal SCC (2, 10). To the best of our knowledge, the prognostic significance of metabolic signature in OSCC remains largely underexplored until now.

Burgeoning development of genome-wide sequencing technology and assembly of The Cancer Genome Atlas (TCGA) and Gene Expression Omnibus (GEO) databases provide rich resources for biomarker development to better early diagnosis, patient stratification, personalized treatment as well as prognostic prediction (11). Based on these datasets, several biomarkers derived from aberrant DNA methylation, mRNA/microRNA/lncRNA expression or combinations of these abnormalities such as gene signatures have been identified and validated to predict survival in patients with OSCC (12–14). For example, a three lncRNA-based signature and a 7 CpG-based signature coupled with gene expression have been successfully established for OSCC prognostic prediction (15, 16).

In the present study, we utilized publicly available RNA sequencing (RNA-seq) data of OSCC samples from TCGA project, constructed and validated a novel prognostic signature based on 5-metabolic pathway-related gene sets by bioinformatics approaches *via* integrating single-sample gene set enrichment analysis (ssGSEA), least absolute shrinkage and selection operator (LASSO) and Cox regression analyses.

## Materials and Methods

### Data Acquisition and Preprocessing

TCGA represents international collaborative efforts to comprehensively delineate the biological characteristics of common human cancers and becomes the rich source to establish the link between genomic features and clinicopathological information among individual types of cancer largely based on the detailed datasets including RNA-seq, demographic information, clinical stage and patient survival (11). The original HNSCC RNA-seq data were downloaded from TCGA database and all OSCC relevant datasets were further retrieved from TCGA-HNSC dataset. A total number of 328 OSCC tumors and 32 normal counterparts were identified and enrolled. All relevant epidemiological, clinicopathological as well as follow-up data were obtained and collated in **Supplementary Table S1**. Moreover, two independent OSCC datasets (GSE41613 and GSE42743) deposited by Chen C’s group in GEO database were found and utilized (14). And “Combat” in R package “sva” was used to remove batch effects. Patient informed consent and approval of the institutional review board were waived given the use of the existing, publically available datasets. Moreover, the immunohistochemical images from the Human Protein Atlas (HPA) database were used to detect the expression of the genes at the translational level (17).

### Acquisition of Metabolism-Related Gene Dataset

These metabolism-related gene datasets were downloaded from the KEGG pathway database (18). There are 90 metabolic pathways including nucleotide metabolism, amino acid metabolism, sand glycan biosynthesis and metabolism. All gene sets of these metabolic pathways were listed in **Supplementary Table S2**.

### Construction of Metabolism-Based Prognostic Signature for OSCC

All OSCC patients from TCGA-HNSC dataset were randomly divided into two cohorts (training cohort: testing cohort=7:3). Datasets from GSE41613 (97) and GSE42743 (74) were pooled and defined as an independent, validation cohort (14). The prognostic signature was constructed according to the following steps (**Supplementary Figure S1**). Firstly, single-sample GSEA (ssGSEA) was used to calculate and standardize the enrichment scores of individual metabolism-related gene set in each sample (19, 20). Secondly, dysregulated metabolic pathways between tumors and normal samples were screened by cut-off FDR<0.05. Thirdly, the univariate Cox regression was used to identify survival-related metabolic pathways with the *P*-value <0.05. Fourthly, the LASSO regression was performed on these survival-related metabolic pathways, and the non-zero coefficients’ metabolic pathways were filtered out for further analyses. Finally, the final metabolism-based prognostic model was constructed by stepwise multivariate Cox regression analysis. The calculation formula for risk scores of five metabolic pathways was as follows: $\text{Risk score}=\sum_{i=1}^{n}\beta i*xi$. *x* was the ssGSEA score for each metabolic pathways and *β* was the regression coefficients in the multivariable Cox regression analysis in the training set All these analyses were performed using the R software (version 3.6.3).

### Statistical Analyses

A receiver operating characteristic (ROC) curve was plotted by “survival ROC” package in R and the optimal cut-off value for risk score was identified using Youden index. All patients were divided into low-risk and high-risk groups based on the cut-off value. The overall survival was counted with Kaplan-Meier method and the difference was compared with log-rank test. A time-dependent ROC curve with 1, 3, 5 years as the defining point was performed to evaluate the predictive value of risk score. The calibration curve was plotted to evaluate the difference between the predicted and actual values of the predictive model. The comparisons of clinicopathological parameters (age, gender, grade, tumor stage and margin status) between high-risk and low-risk groups were analyzed *via* Chi-square test. The evaluation of statistically significant differences of metabolic pathways risk score between subgroups was performed by one-way ANOVA analyses. Univariate and multivariate Cox regression analyses were employed to determine prognostic factors associated with survival. All these analyses were performed using the R software (version 3.6.3). All statistical tests were two sided and *P* values less than 0.05 were considered statistically significant.

## Results

### Patient Cohorts and RNA-Seq Datasets

After data screening and filtering, 328 OSCC samples and 32 normal samples with original RNA-seq data and clinical follow-up information from TCGA were retrieved and randomly divided into training and testing cohorts. RNA-seq data and clinical information of 171 OSCC samples (defined as validation cohort) were obtained from GSE41613 and GSE42743 (14).

### Identification of Dysregulated Metabolic Pathways and Construction of Metabolic Prognostic Signature in OSCC

**Figure 1:**
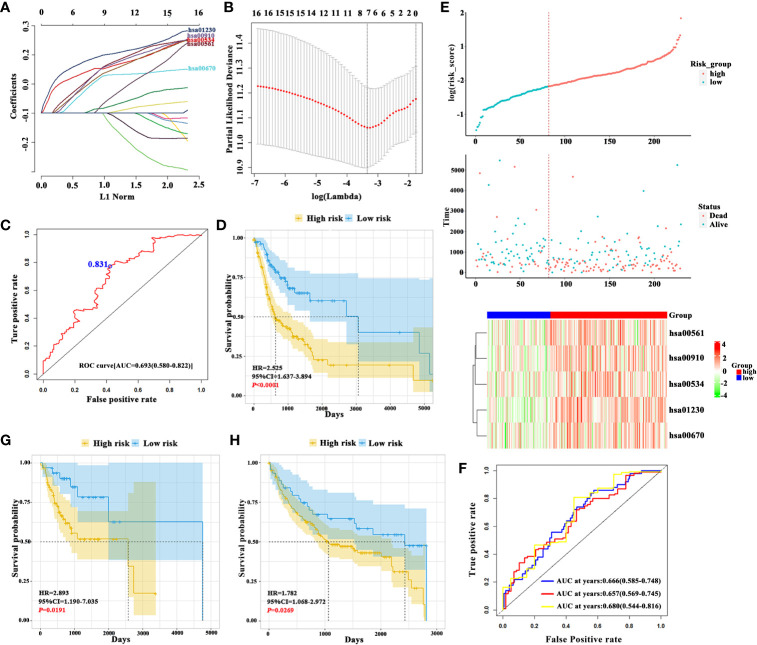
Construction of 5-metabolic pathways signature and its prognostic value **(A)**. The coefficient profile plot of 16 survival-related metabolic pathways was produced against the log lambda sequence **(B)**. Tuning parameter (lambda) selection in the LASSO model used tenfold cross-validation *via* minimum criteria. Dotted vertical lines were drawn at the optimal values using the minimum criteria and the 1 standard error of the minimum criteria (the 1-SE criteria) **(C)**. ROC analysis of sensitivity and specificity of overall survival time by the 5-metabolic pathways signature based risk score. The blue dot represents the optimal cut-off value in training cohort using ROC analysis **(D)**. The Kaplan-Meier analyses revealed significant associations between 5MPS and OS in patients from the training cohort **(E)**. From top to bottom: The survival status of patients with OSCC in training cohort. Patient subgroups with high and low-risk score were classified by the optimal cut-off value. The heatmap show the distribution of 5 metabolic pathways enrichment score in high risk and low risk groups in training cohort **(F)**. The time-dependent ROC curve analysis with 1, 3, 5 years as the defining point was performed to evaluate the predictive value of the 5-metabolic pathways risk score in training cohort **(G, H)**. The Kaplan-Meier analyses revealed significant associations between 5MPS and OS in patients from the testing **(G)** and validation cohorts **(H)**.

The detailed analytic pipeline for signature development was shown in **Supplementary Figure S1**. Initially, we determined enrichment scores of 90 known metabolic pathways in the training datasets by ssGSEA and assigned individual score for every metabolic pathways in each sample. Then, we identified 75 dysregulated metabolic pathways which significantly differed between OSCC and normal samples with FDR<0.05 and 16 survival-related metabolic pathways by univariate Cox regression analysis (*P*<0.05) were found. As shown in **Figures 1A, B**, 7 survival-related metabolic pathways were selected by LASSO regression for further stepwise multiple Cox regression analyses. Finally, we developed a prognostic signature consisting of 5 metabolic pathways: hsa00561 (Glycerolipid metabolism), hsa00910 (Nitrogen metabolism), hsa00534 (Glycosaminoglycan biosynthesis-heparan sulfate/heparin), hsa01230 (Biosynthesis of amino acids) and hsa00670 (One carbon pool by folate). The coefficients of 5 metabolic pathways were shown: hsa00561: 0.22332, hsa00910: 0.18382, hsa00534: 0.20845, hsa01230: 0.33467, and hsa00670: 0.19317.

### The 5-Metabolic Pathways Signature Predicts Survival of Patients With OSCC

The risk score formula was developed based on enrichment score and coefficients of these 5 metabolic pathways, which was named as 5-metabolic pathways signature (5MPS). An optimal cut-off value was selected as 0.831 with maximal sensitivity and specificity as evidenced by AUC 0.693 (**Figure 1C**). All patients in the training sets were divided into high-risk group and low-risk group according to this optimal cut-off value. The Kaplan-Meier analyses indicated that patients in high-risk subgroup had a worse prognosis than those in low-risk subgroup (*P*<0.0001, **Figures 1D, E**). Moreover, a time-dependent receiver operating characteristic (ROC) curve was performed to estimate the sensitivity and specificity of this 5MPS. Data listed in **Figure 1F** indicated that this signature to predict patient survival in 1, 3 and 5 years was satisfactory with AUC 0.666, 0.657, and 0.680 in the training cohort. In addition, the calibration curve was plotted to evaluate the performance of this signature. And the result shown in **Supplementary Figure S2** revealed that the prediction was close to a 45 degree slash, thus indicating that 5MPS prediction was well consistent with actual observation.

### Validation of 5MPS Predictive Values in Multiple Independent OSCC Cohorts

Patients in testing and validation cohorts were stratified into high-risk and low-risk subgroups according to the 5MPS. As shown in **Figures 1G, H**; **Supplementary Figure S3**, the results from Kaplan-Meier analyses indicated that patients in high-risk subgroup had a lower OS ratio than those in low-risk subgroup in testing cohort (*P*=0.0191), validation cohort (*P*=0.0269) and TCGA-OSCC cohort (*P*<0.0001). As shown in **Supplementary Figure S4**, the AUC from time-dependent ROC curves were 0.702, 0.618, and 0.626 in testing cohort and 0.616, 0.628 and 0.688 in validation cohort, respectively.

### Correlations Between 5MPS With Clinicopathological Characteristics in OSCC

**Figure 2:**
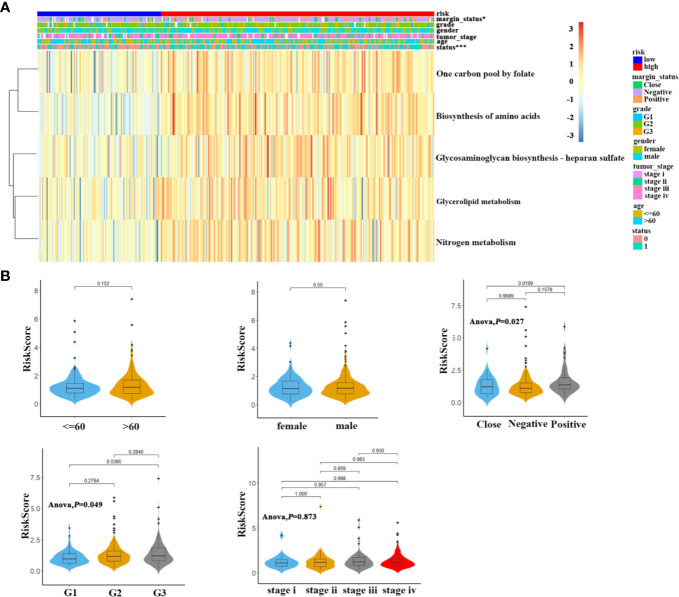
The association of 5-metabolic pathways risk score and clinicopathological parameters **(A)**. The heatmap shows the distribution of 5 metabolic pathways enrichment score and clinicopathological parameters between high risk and low risk groups in TCGA OSCC cohort **(B)**. Distribution of 5-metabolic pathways risk score in different subgroups was compared, which stratified by age, gender, margin status, grade and tumor stage.

As shown in **Figure 2A**, the heatmap showed the distributions of enrichment scores and several clinicopathological parameters including age, gender, grade, margin status, tumor stage between patients in high- and low-risk subgroups. We found that 5MPS was significantly associated with margin status (*P*<0.05) and survival status (*P*<0.0001). Moreover, as shown in **Figure 2B**, patents stratified with differentiation degree or margin status had significantly different risk scores, which indicated higher risk score was associated with higher pathological grade and positive margin status.

### Univariate and Multivariate Cox Regression Analyses for 5MPS

**Figure 3:**
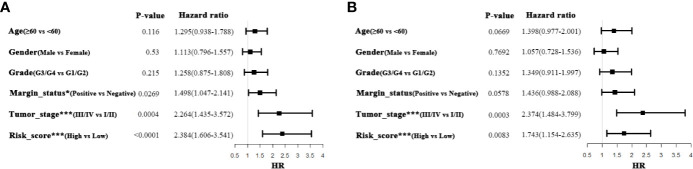
Univariate and Multivariate Cox-regression analyses of 5-metabolic pathways signature and clinicopathological parameters in TCGA OSCC cohort (**A** Univariate analysis; **B** Multivariate analysis).

**Figure 4:**
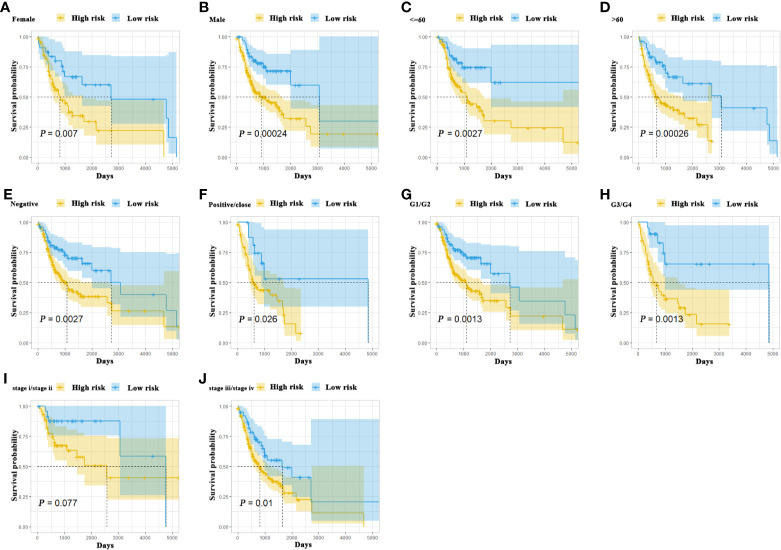
The Kaplan-Meier analyses revealed significant associations between 5MPS and OS of OSCC patients in different clinical subgroups, grouping based on their age, gender, margin status, grade, and tumor stage **(A-J)**.

To further delineate the prognostic value of 5MPS in OSCC, we performed univariate and multivariate Cox regression using the whole OSCC cohort from TCGA-HNSC dataset. Our data revealed that 5MPS was associated with overall survival in OSCC patients (HR, 2.384; 95% CI: 1.606–3.541; *P <*0.0001). In addition, some well-established clinicopathological parameters such as margin status and tumor stage were also identified to be prognostic. Moreover, we performed multivariate Cox regression analyses to eliminate confounding factors and highlight the prognostic value of 5MPS. As shown in **Figure 3**, 5MPS was identified as an independent predictive factor for OSCC (HR, 1.743; 95% CI: 1.154-2.635; *P* =0.0083). Consistently, as shown in **Figures 4A–J**, this 5MPS robustly predicted overall survival in OSCC patients stratified by epidemiological, clinical and pathological parameters.

### The Representative Genes in Five Metabolic Pathways Predict Survival in Patients With OSCC

**Figure 5:**
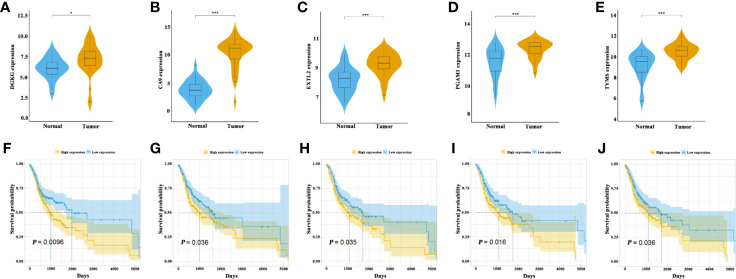
The association of five metabolism-related genes from these five metabolic pathways with OS in OSCC patients **(A–E)**. The expression of DGKG, CA9, EXTL2, PGAM1 and TYMS were compared between tumor and normal **(F–J)**. The Kaplan-Meier analyses revealed significant associations between DGKG **(F)**, CA9 **(G)**, EXTL2 **(H)**, PGAM1 **(I)** and TYMS **(J)** and OS of OSCC patients in TCGA OSCC cohort, *P < .05,***P < .001.

To further characterize these 5 metabolic pathways integrated into 5MPS, we selected representative genes such as DGKG (Diacylglycerol kinase gamm), CA9 (Carbonic anhydrase 9), EXTL2 (Exostosin like glycosyltransferase 2), PGAM1 (Phosphoglycerate mutase 1), TYMS (Thymidylate Synthase) from these dysregulated metabolic pathways. The expression of these 5 genes was compared between 32 paired tumor and normal samples from TCGA OSCC cohort. As shown in **Figures 5A–E**, expression levels of these genes were significantly higher in OSCC as compared to their normal counterparts. Consistently, as shown in **Supplementary Figure S5**, we retrieved the data from the human protein atlas (HPA) platform and found that the protein abundance of these 5 genes in HNSCC samples appeared to be upregulated relative to their healthy counterparts based on both intensity and quantity of staining, although we can’t definitively perform these comparisons by statistical analyses due to the lack of detailed staining data (17).

Moreover, we divided patients into low and high expression subgroups based on the median values of gene expression. Kaplan-Meier analyses indicated that these five genes were significantly associated with OS in OSCC patients (**Figures 5F–J**).

## Discussion

OSCC is a lethal malignancy characterized by rapid progression, cervical lymph node involvement and relatively high mortality. However, the traditional prognostic parameters fail to fully satisfy the clinical demands for accurate and individualized prognostic evaluation (2). Therefore, it is imperative to develop more accurate and sensitive biomarkers for OSCC diagnosis and prognostic prediction. Here, we constructed and validated a novel prognostic biomarker namely 5-metabolic pathways signature (5MPS) for OSCC *via* an integrative bioinformatics approach.

Most metabolic processes such as those involving energy and amino acid catabolism are common and pivotal to all living cells. However, compared to normal cells, some metabolic pathways such as glycolysis and fatty-acid oxidation have undergone tremendous changes in cancer cells due to their high energy requirements to support highly proliferation and metastasis. Recently, cancer metabolomics has been proposed to comprehensively characterize hallmarks of cancer-related metabolic changes (21). However, most of previous studies have largely focused on individual metabolic pathway underlying oral tumorigenesis and identified multiple biomarkers with diagnostic and prognostic significance as well as potential therapeutic targets (22, 23). Here, we exploited an integrative bioinformatics approach to comprehensively characterize the survival-related metabolic changes in OSCC. By utilizing RNA-seq datasets from TCGA and GEO, we developed a novel prognostic signature based on 5 dysregulated metabolic pathways in OSCC.

Cancer metabolomics have provided sensitive and thorough metabolic signatures as effective biomarkers for cancer diagnosis, treatment and prevention (24). Comprehensive analyses of metabolites in clinical samples such as saliva, urine and serum have identified valuable biomarkers for cancer diagnosis and prognostic prediction in multiple cancers including OSCC (25–28). Deregulated metabolic pathways including glucose metabolism, glutaminolysis and tricarboxylic acid cycle have been reported in OSCC (26). However, these abovementioned studies largely focused on a few metabolites or individual metabolites in cancer. Here, we constructed an integrative signature based several metabolic gene sets in OSCC. This analytic approach enabled simultaneous integration of dysregulated metabolic pathways that had impact on patient prognosis. As expected, our 5MPS robustly stratified patients into subgroups and served as an independent factor affecting patient survival. We believe that this 5MPS will be beneficial for predicting patient survival when it is added into current clinical regime. Of course, to reinforce its translational potentials, the predictive performance of 5MPS should be further validated.

Beyond this prognostic value of 5MPS, our 5MPS also reflected the importance of these dysregulated metabolic pathways involved in OSCC tumorigenesis. These aberrant metabolic pathways provided nutrients and biomass to meet the high energy and biosynthetic demand of cancer cells (21). For example, glycerolipid metabolism and fatty-acid oxidation have undergone tremendous changes in cancer cells to support their proliferation and metastasis (29, 30). In addition, oncogene-driven activation of cell growth was associated with increased amino acid uptake and biosynthesis (31). Folates promoted one-carbon metabolism essential for purines and thymidylate biosynthesis and enhanced DNA replication in cancer cells (32). In line with these, our results from 5 representative genes in these pathways showed that they were significantly upregulated in OSCC relative to their normal counterparts and associated with unfavorable survival. Previous studies have reported that CA9, EXTL2, PGAM1, and TYMS were dysregulated across multiple human cancers and intricately associated with tumorigenesis by functioning as key enzymes underlying metabolism (33–36). Moreover, high expression of CA9 and PGAM1 were associated with inferior prognosis in OSCC, which in part strengthened our data (33, 35). Noticeably, Yang et al. utilized gas chromatography-mass spectrometry high-throughput analysis to determine the amino acid metabolic characteristics of OSCC and found that a panel including three amino acids (glutamate, aspartic acid, and proline) was identified as potential diagnostic biomarkers of OSCC (37). These findings may add further support to the key roles of dysregulated metabolism responsible for OSCC initiation and progression, thus ultimately impacting patient prognosis.

Although, 5MPS identified here was robust and promising, there were still several limitations. Firstly, the number of OSCC samples is relatively small. This signature is still needed to be independently validated in more cohorts. However, the data from multiple databases and our training-testing-validation cohort design might compensate for this disadvantage. Secondly, 5MPS mainly depended on RNA-seq data whose procedures of detection, quantification should be standardized and normalized. Thirdly, 5MPS was based on gene sets so that might limit its clinical application to a certain degree.

## Conclusion

In conclusion, our study identified a novel 5-metabolic pathways signature (5MPS) based on RNA-seq data which reflected dysregulated metabolic pathways and their prognostic significance in OSCC. This 5MPS served as a novel prognostic biomarker for OSCC, which was warranted to be validated further in a large amount of prospectively enrolled patients.

## Data Availability Statement

The datasets presented in this study can be found in online repositories. The names of the repository/repositories and accession number(s) can be found in the article/**Supplementary Material**.

## Author Contributions

XW and YY performed the data collection, analysis and manuscript writing. ZL, HG, and DW assisted out data collection and statistical analyses. YW conceived and supervised the whole project. All authors contributed to the article and approved the submitted version.

## Funding

This work is financially supported, in whole or in part, by National Natural Science Foundation of China (81572669, 81602386, 81602378), Natural Science Foundation of Jiangsu Province (BK20161564, BK20161024), A Project Funded by the Priority Academic Program Development of Jiangsu Higher Education Institutions (2018-87), Research grant from Nanjing Medical University and Southeast University (2017DN20) and Project from Nanjing Municipal Committee of Science and Technology (201803044). The funders had no role in study design, data collection, data analysis, interpretation, or writing of the report.

## Conflict of Interest

The authors declare that the research was conducted in the absence of any commercial or financial relationships that could be construed as a potential conflict of interest.

## Supplementary Material

Supplementary Figure 1 The procedures for analyzing 5MPS in oral squamous cell carcinoma (OSCC).

Supplementary Figure 2 The calibration curve was plotted to evaluate 5-metabolic pathways signature predictive performance for predicting OS of patients at 1, 3, 5 years in training cohort.

Supplementary Figure 3 The Kaplan-Meier analysis revealed significant associations between 5-metabolic pathways signature and OS in patients from the TCGA OSCC cohort.

Supplementary Figure 4 The time-dependent ROC curve analysis with 1, 3, 5 years as the defining point was performed to evaluate the predictive value of the 5-metabolic pathways risk score in testing cohort **(A)** and validation cohort **(B)**.

Supplementary Figure 5 The immunohistochemical images from the Human Protein Atlas (HPA) database were used to compare the expression of DGKG, CA9, EXTL2, PGAM1 and TYMS at the translational level between HNSCC and normal samples. Both intensity and quantity of immunohistochemical staining in HNSCC and Normal oral mucosa were retrieved and shown in the right panel for each gene of interest.
