# Supplementary figures and images for: Identification of a Transcriptional Prognostic Signature From Five Metabolic Pathways in Oral Squamous Cell Carcinoma
Source: Front Oncol. 2020 Dec 2;10:572919. doi: 10.3389/fonc.2020.572919 (PMC7793793; doi:10.3389/fonc.2020.572919)

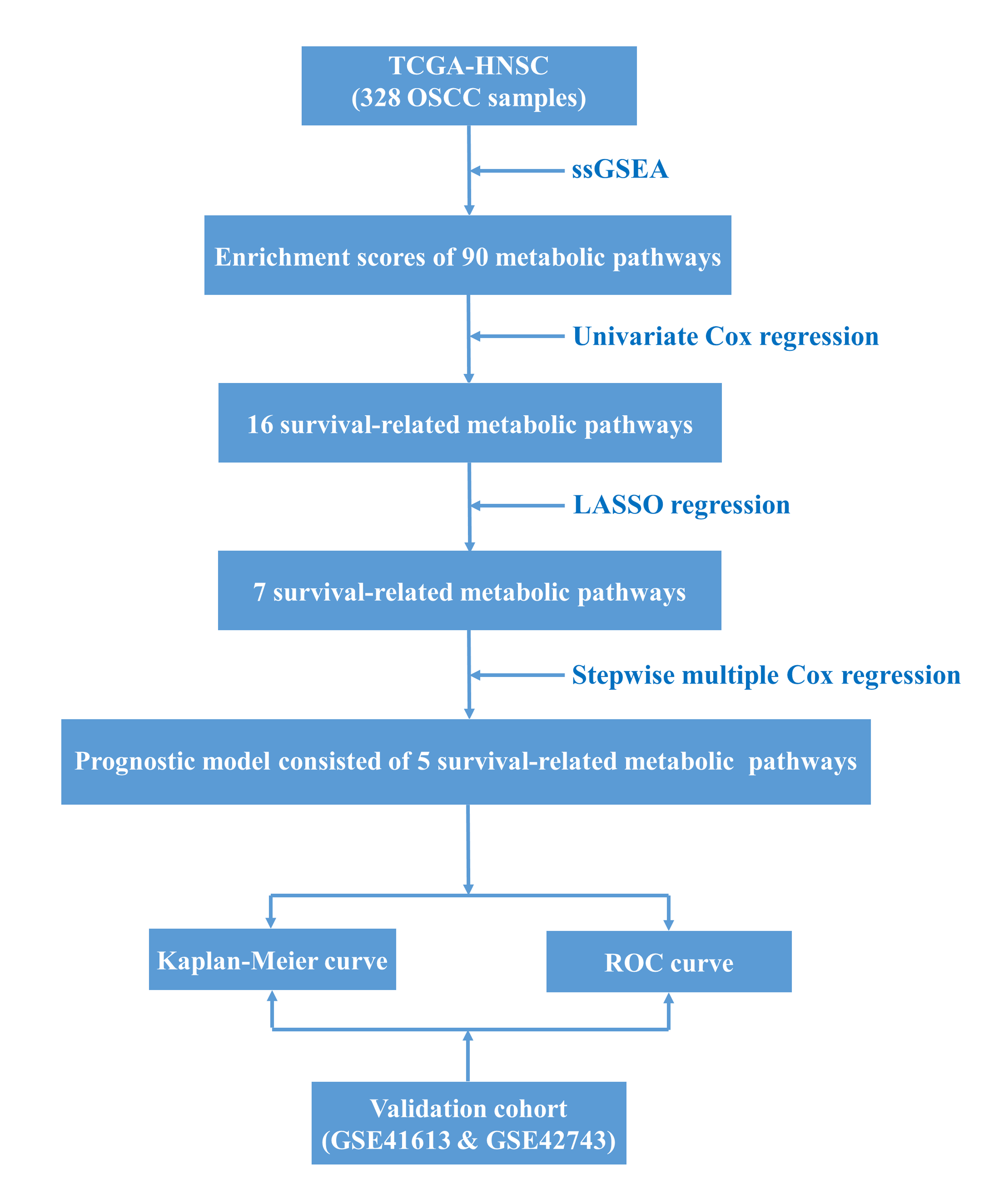

Supplement: Supplementary Figure 1 — The procedures for analyzing 5MPS in oral squamous cell carcinoma (OSCC). [file Image_1.tif]

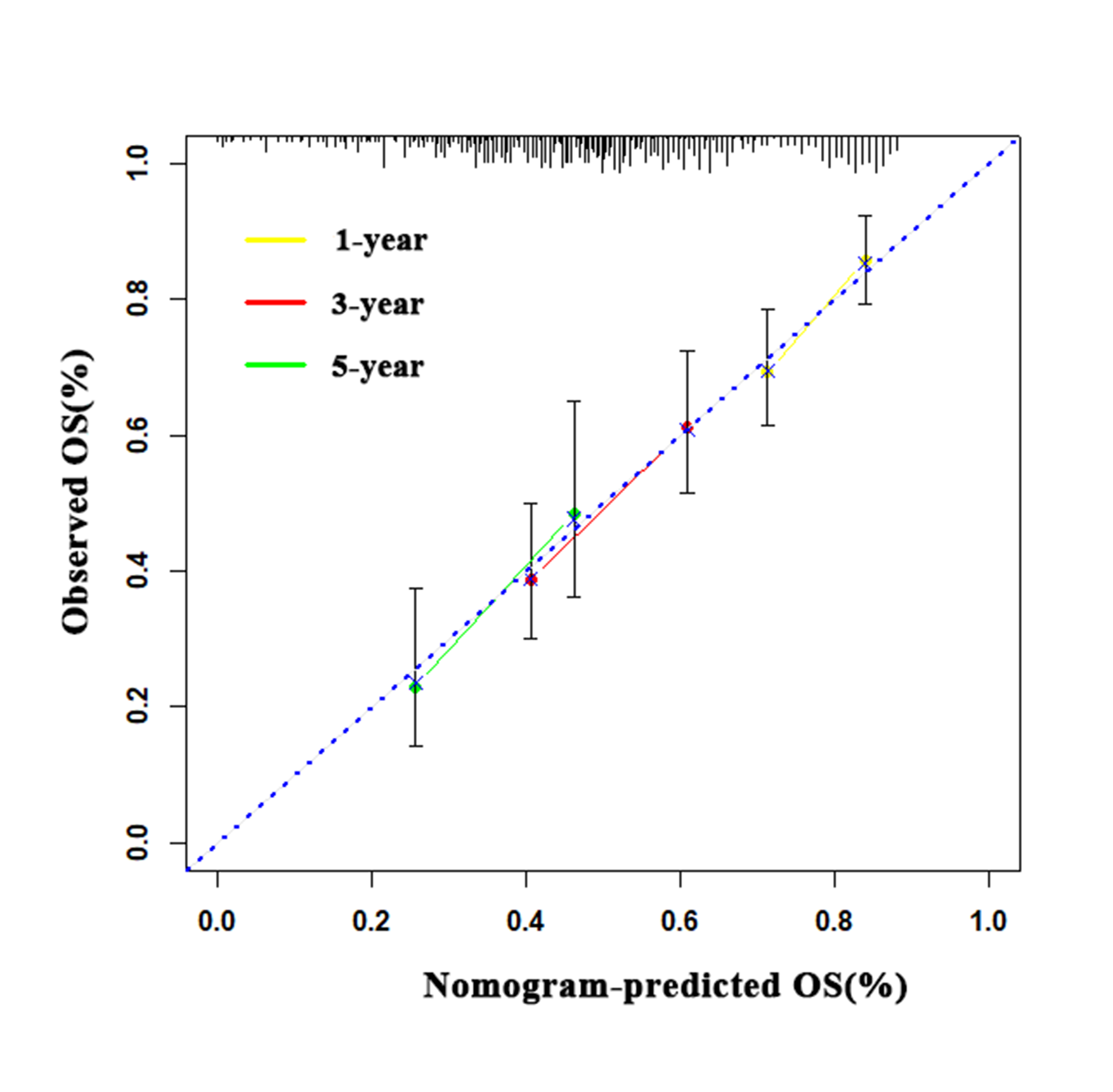

Supplement: Supplementary Figure 2 — The calibration curve was plotted to evaluate 5-metabolic pathways signature predictive performance for predicting OS of patients at 1, 3, 5 years in training cohort. [file Image_2.tif]

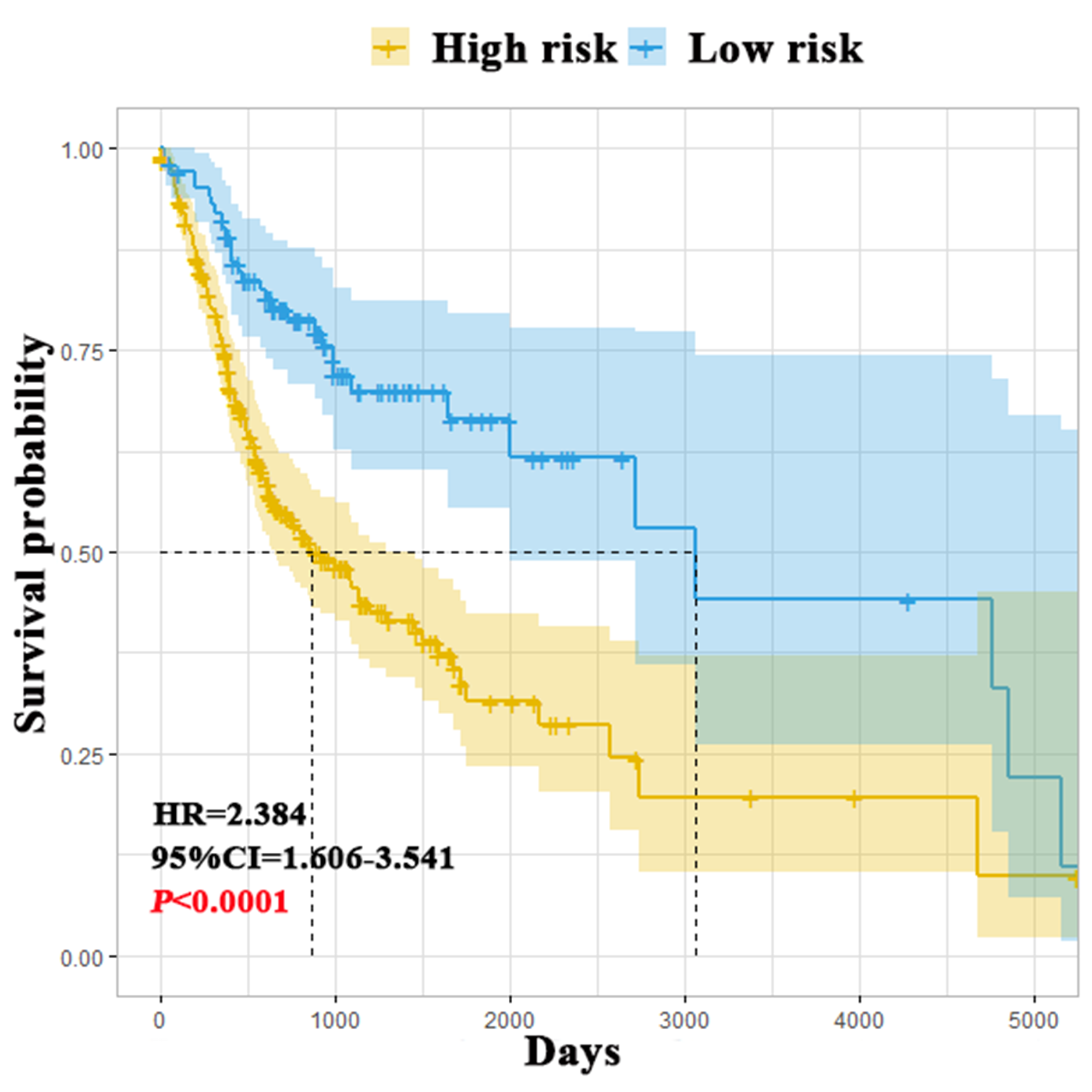

Supplement: Supplementary Figure 3 — The Kaplan-Meier analysis revealed significant associations between 5-metabolic pathways signature and OS in patients from the TCGA OSCC cohort. [file Image_3.tif]

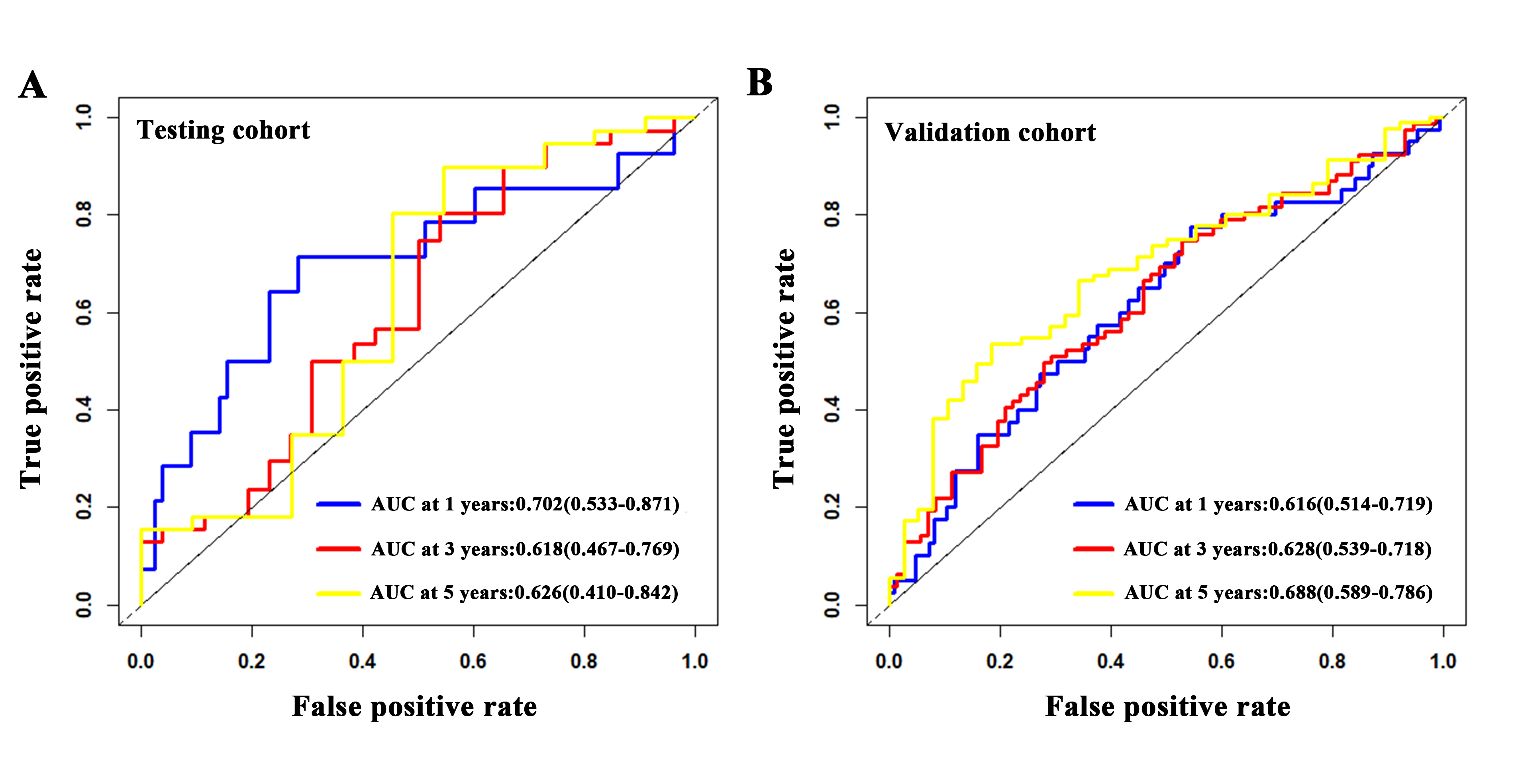

Supplement: Supplementary Figure 4 — The time-dependent ROC curve analysis with 1, 3, 5 years as the defining point was performed to evaluate the predictive value of the 5-metabolic pathways risk score in testing cohort (A) and validation cohort (B). [file Image_4.tif]

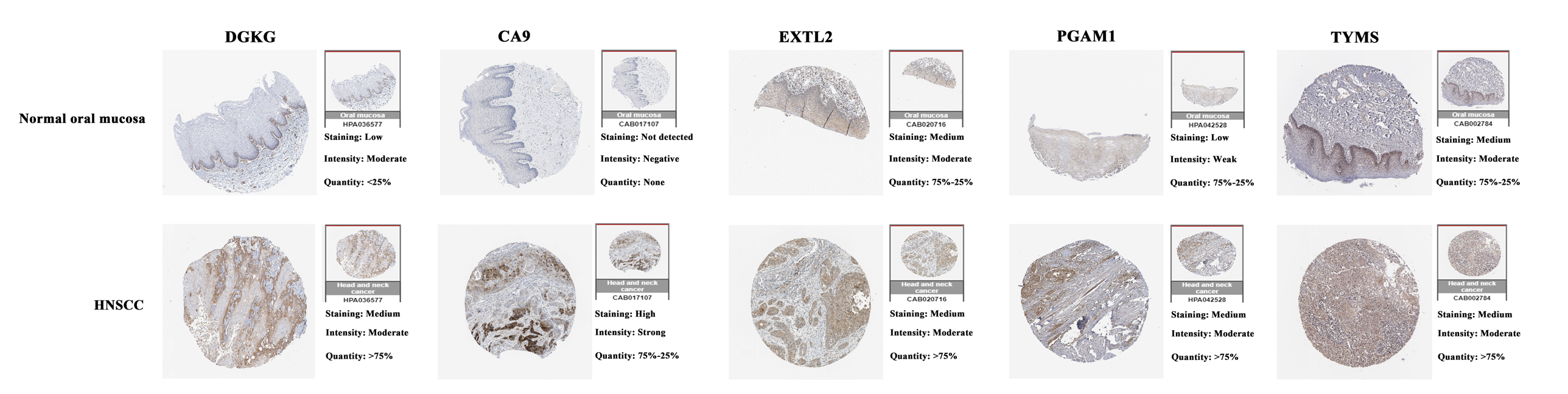

Supplement: Supplementary Figure 5 — The immunohistochemical images from the Human Protein Atlas (HPA) database were used to compare the expression of DGKG, CA9, EXTL2, PGAM1 and TYMS at the translational level between HNSCC and normal samples. Both intensity and quantity of immunohistochemical staining in HNSCC and Normal oral mucosa were retrieved and shown in the right panel for each gene of interest. [file Image_5.tif]
